# Persistent High IgG Phase I Antibody Levels against *Coxiella burnetii* among Veterinarians Compared to Patients Previously Diagnosed with Acute Q Fever after Three Years of Follow-Up

**DOI:** 10.1371/journal.pone.0116937

**Published:** 2015-01-20

**Authors:** Cornelia C. H. Wielders, Anneroos W. Boerman, Barbara Schimmer, René van den Brom, Daan W. Notermans, Wim van der Hoek, Peter M. Schneeberger

**Affiliations:** 1 Department of Medical Microbiology and Infection Control, Jeroen Bosch Hospital, ’s-Hertogenbosch, The Netherlands; 2 Centre for Infectious Disease Control, National Institute for Public Health and the Environment (RIVM), Bilthoven, The Netherlands; 3 Department of Small Ruminant Health, GD Animal Health, Deventer, The Netherlands; University of Arkansas for Medical Sciences, UNITED STATES

## Abstract

**Background:**

Little is known about the development of chronic Q fever in occupational risk groups. The aim of this study was to perform long-term follow-up of *Coxiella burnetii* seropositive veterinarians and investigate the course of IgG phase I and phase II antibodies against *C. burnetii* antigens and to compare this course with that in patients previously diagnosed with acute Q fever.

**Methods:**

Veterinarians with IgG phase I ≥1:256 (immunofluorescence assay) that participated in a previous seroprevalence study were asked to provide a second blood sample three years later. IgG antibody profiles were compared to a group of acute Q fever patients who had IgG phase I ≥1:256 twelve months after diagnosis.

**Results:**

IgG phase I was detected in all veterinarians (n = 76) and in 85% of Q fever patients (n = 98) after three years (p<0.001). IgG phase I ≥1:1,024, indicating possible chronic Q fever, was found in 36% of veterinarians and 12% of patients (OR 3.95, 95% CI: 1.84–8.49).

**Conclusions:**

IgG phase I persists among veterinarians presumably because of continuous exposure to *C. burnetii* during their work. Serological and clinical follow-up of occupationally exposed risk groups should be considered.

## Introduction

Q fever is a zoonotic disease caused by the intracellular, Gram-negative bacterium *Coxiella burnetii*. Cattle, sheep, and goats are considered the primary source of infection [1]. Infected animals can shed the bacterium in milk, feces, urine, and especially in birth products [2]. The main route of transmission to humans is by inhalation of *C. burnetii* contaminated aerosols [3]. Acute Q fever usually presents as a flu-like illness, pneumonia or hepatitis. However, in 60% of the cases the primary infection remains asymptomatic [3]. From 2007 until 2010, the Netherlands experienced the largest community Q fever epidemic documented in the world, which resulted in >4,000 notified patients [4].

The Dutch epidemic has passed and priorities shifted from acute Q fever to chronic Q fever [5]. Development of chronic Q fever, mostly presenting as endocarditis or vascular infections [6,7], has been reported in the literature in an estimated 2% of acute Q fever patients [8]. Clinical risk factors for chronic Q fever development are heart valve disease, vascular aneurysms or grafts, immunosuppression, pregnancy, and renal disease [1,9,10].

*Coxiella burnetii* has two antigenic phases: during acute infection IgM and IgG antibodies against phase II *C. burnetii* antigens predominate, while a persisting high titer of IgG antibodies against phase I is suspect for chronic infection [2]. There is no international consensus of the diagnostic criteria of chronic Q fever and defining chronic Q fever is still under debate [11–13]. The Dutch Q fever Consensus Group established a case definition of chronic Q fever and classified it into proven, probable, and possible [11]. Especially for the possible chronic Q fever cases (IgG phase I ≥1:1,024 and no symptoms or risk factors) it is unclear whether they represent true chronic cases with intracellular persistence of *C. burnetii*.

In 2009, a cross-sectional study among Dutch livestock veterinarians estimated a seroprevalence of 65.1% based on presence of *C. burnetii* IgG phase II antibodies (cut-off IgG phase I and phase II ≥1:32 or solitary IgG phase II ≥1:512) [14]. In 2010, a similar study was targeted at veterinarians working with companion animals. Other seroprevalence studies conducted in the Netherlands among occupationally exposed persons showed high estimates as well: 73.5% in dairy goat farmers, and 66.7% and 51.3% in dairy and non-dairy sheep farmers, respectively [15,16]. In other countries, seroprevalence rates of 22.2% (United States) [17] and 38.2% (Germany) [18] have been described among veterinarians. Despite these high seroprevalence rates, follow-up serology has rarely been described in occupational groups, and a proper assessment of their risk for chronic Q fever development is unknown.

Therefore, aim of this study was: (i) to describe the course of *C. burnetii* IgG phase I and II antibodies in veterinarians over a three-year period and compare this course with that in acute Q fever patients who were diagnosed four years before, and (ii) to investigate factors associated with constant or increasing IgG phase I titers during follow-up to improve recommendations for prevention and early diagnosis of chronic Q fever in this occupational group.

## Materials and Methods

### Ethics statement

This study was approved by the Medical Ethical Committee Brabant (METC Brabant, reference NL35654.028.11). Written informed consent was obtained from all participants included in this study.

### Study design and population

**Veterinarians**. Two cross-sectional studies among Dutch veterinarians were carried out in November 2009 (livestock veterinarians) and April 2010 (companion animal veterinarians) in order to assess the *C. burnetii* seroprevalence including risk factors for seropositivity in this occupational group. A total of 432 Dutch veterinarians and veterinary students in their final year of studies completed a questionnaire and provided a serum sample. The study design of the cross-sectional study in 2009 has been described before [14].

All veterinarians with an IgG phase I titer ≥1:256 who participated in one of the two previous studies were invited for a follow-up study (three to four years after first sample). Participation consisted of completing a questionnaire and providing a single blood sample after giving written informed consent. The questionnaire consisted of four sections with questions pertaining to personal demographic characteristics, general medical history, acute Q fever-history, subjective health status (EQ-5D) [19] and complaints of fatigue (part of the Nijmegen Clinical Screening Instrument [NCSI] [20]). Non-responders received a written reminder four weeks after the first invitation. Information about occupational exposures were extracted from the questionnaire data collected during the previous cross-sectional studies in 2009 and 2010, including details on type of veterinary practice, years of clinical veterinary practice and weekly contact with livestock, companion animals and animal related products.

All participating veterinarians received their individual laboratory results accompanied with an assessment by a medical microbiologist of the Regional Laboratory for Medical Microbiology (LMM) of the Jeroen Bosch Hospital (JBH), ’s-Hertogenbosch about the likelihood of having chronic Q fever and the need for further medical assessment.

**Q fever patients** Patients diagnosed with acute Q fever in 2007 or 2008 by the LMM of JBH as part of normal patient care and who participated in a four-year follow-up study (performed in 2011 and 2012), were included as reference group. This serological follow-up study was performed among adult Q fever patients whose antibody levels were tested approximately twelve months after the acute Q fever diagnosis as well, as part of routine patient follow-up. The same questionnaire as described above was used. For the current study, we only included patients with IgG phase I titer ≥1:256 at twelve-month follow-up after acute Q fever diagnosis. We choose to use this sample as it is unknown when the veterinarians were infected with *C. burnetii* and in this way follow-up times were comparable for veterinarians and Q fever patients.

### Laboratory methods

At follow-up, both the veterinarians and the patient reference group were tested for presence of *C. burnetii* IgG phase I and phase II antibodies using indirect immunofluorescence assay (IFA; Focus Diagnostics, Inc., Cypress, CA, USA) in serum. An IgG phase I and/or phase II titer of ≥1:32 was defined as positive and the end-point titration was established by serial two-fold dilutions of the serum. Additionally, all serum samples of veterinarians were tested for *C. burnetii* DNA by PCR assay (in-house assay) [21,22], while for the reference group this was only done when IgG phase I was ≥1:512.

### Data analysis

Veterinarians and Q fever patients who did not submit both a blood sample and questionnaire were excluded from the analysis. Statistical analyses were carried out using IBM SPSS for Windows version 19.0.0 (SPSS Inc., Armonk, NY, USA). For descriptive characteristics, relative frequencies and the medians with interquartile ranges (IQRs) were calculated. To determine difference in serological results between veterinarians and Q fever patients, the Mann-Whitney U test was used. Variables with a *p*-value of <0.05 were considered statistically significant. The occurrence of IgG phase I titers ≥1:1,024 among veterinarians and Q fever patients was compared and an odds ratio (OR) and 95% confidence interval (95% CI) was calculated. Univariate analyses were performed among veterinarians to investigate possible associations between persisting IgG phase I titers and occupational and non-occupational exposures and relative risks (RR) with 95% CI were calculated.

## Results

Of the 98 veterinarians with an IgG phase I titer ≥1:256 in the previous serological studies, 79 (80.6%) participated in this follow-up study. Three veterinarians were excluded in further analyses because of incomplete data, thus, 76 (77.6%) veterinarians remained for the analysis. For the Q fever patient group, 149 of 741 (20.1%) patients were selected based on the presence of IgG phase I ≥1:256 at twelve months after acute Q fever diagnosis. Of these, 51 patients were excluded because of not participating in the four-year follow-up study. In total, 98 patients (65.8% of patients with an IgG phase I titer ≥1:256) were included. The median time between the two samples was 39 months (IQR 35–40) for the veterinarians and 35 months (IQR 34–39) for the Q fever patients, which differed significantly (*p*<0.001). From now on we call this an overall three-year follow-up period for both groups.

Veterinarians were younger and more often male than Q fever patients (Table 1). Most of the veterinarians (95.8%) did not recall any symptoms associated with an acute Q fever episode in their medical history. In general, veterinarians had less comorbidity and reported a better health status than Q fever patients.

**Table 1:** Descriptive characteristics of the study population.

**Population characteristics**		**Veterinarians (n = 76)**	**Q fever patients (n = 98)**
		**n (%)**	**n (%)**
Median age^a^ [IQR]		50 [44.3–56.0]	57.5 [50.8–66.0]
Male gender		55 (72.4)	52 (53.1)
Symptoms of acute Q fever^b^		3 (4.2)^f^	91 (92.9)
Antibiotic treatment for Q fever >1 month^b^		0 (0.0)^g^	30 (30.6)
IgG phase I titer first sample	<1:1,024	57 (75.0)	76 (77.6)
	≥1:1,024	19 (25.0)	22 (22.4)
Province of residence	Noord-Brabant (main epidemic area)	10 (13.2)	95 (96.9)
	Other provinces	66 (86.8)	3 (3.1)
Comorbidity	Cardiovascular comorbidity^c^	0 (0.0)	30 (30.9)^g^
	Rheumatic disease	1 (1.3)	9 (9.2)
	Inflammatory bowel disease	1 (1.3)	0 (0.0)
	Diabetes	2 (2.6)	9 (9.2)
	Chronic kidney failure	0 (0.0)	2 (2.0)
	Cancer	1 (1.3)	9 (9.2)
Use of medication past five years	Immunosuppressive medication	1 (1.3)	3 (3.1)
	Cardiovascular medication^d^	5 (6.6)	48 (49.0)
EQ-5D score^e^	Mobility: problems reported	4 (5.3)	31 (32.0)^f^
	Self-care: problems reported	0 (0.0)	2 (2.1)^f^
	Usual activities: problems reported	9 (12.0)^f^	43 (43.9)
	Pain/discomfort: problems reported	15 (19.7)	49 (50.0)
	Anxiety/depression: problems reported	8 (10.5)	21 (21.4)
Median VAS score current health status (range)		83.5 (45–100)^h^	70 (22–100)
Fatigue measured by NCSI	Score <27 (normal)	47 (70.1)^i^	31 (33.0)^j^
	Score 27–35 (mildly affected)	12 (17.9)^i^	14 (14.9)^j^
	Score >35 (severely affected)	8 (11.9)^i^	49 (52.1)^j^

EQ-5D: a standardized instrument for use as a measure of health outcome [19]; IQR: interquartile range; NCSI: Nijmegen Clinical Screening Instrument; VAS: visual analogue scale.

^a^ Age at date of collection follow-up sample.

^b^ Answered with ‘yes’ is shown, ‘no or unknown’ was answered by the remaining participants.

^c^ Pathology of vessels (exclusion of hypertension and varices) or cardiac valves; myocardial infarction, percutaneous coronary intervention, coronary stent, bypass surgery or pacemaker.

^d^ Antihypertensive agents, rhythm and rate control drugs, cholesterol-lowering medicines, anticoagulants, antiplatelet agents.

^e^ EQ-5D score: score 2 and 3 (on a scale of 1–3) defined as problems reported.

^f^ Information missing from five cases.

^g^ Information missing from one case.

^h^ Information missing from three cases.

^i^ Information missing from seven cases.

^j^ Information missing from four cases.

**Figure 1:**
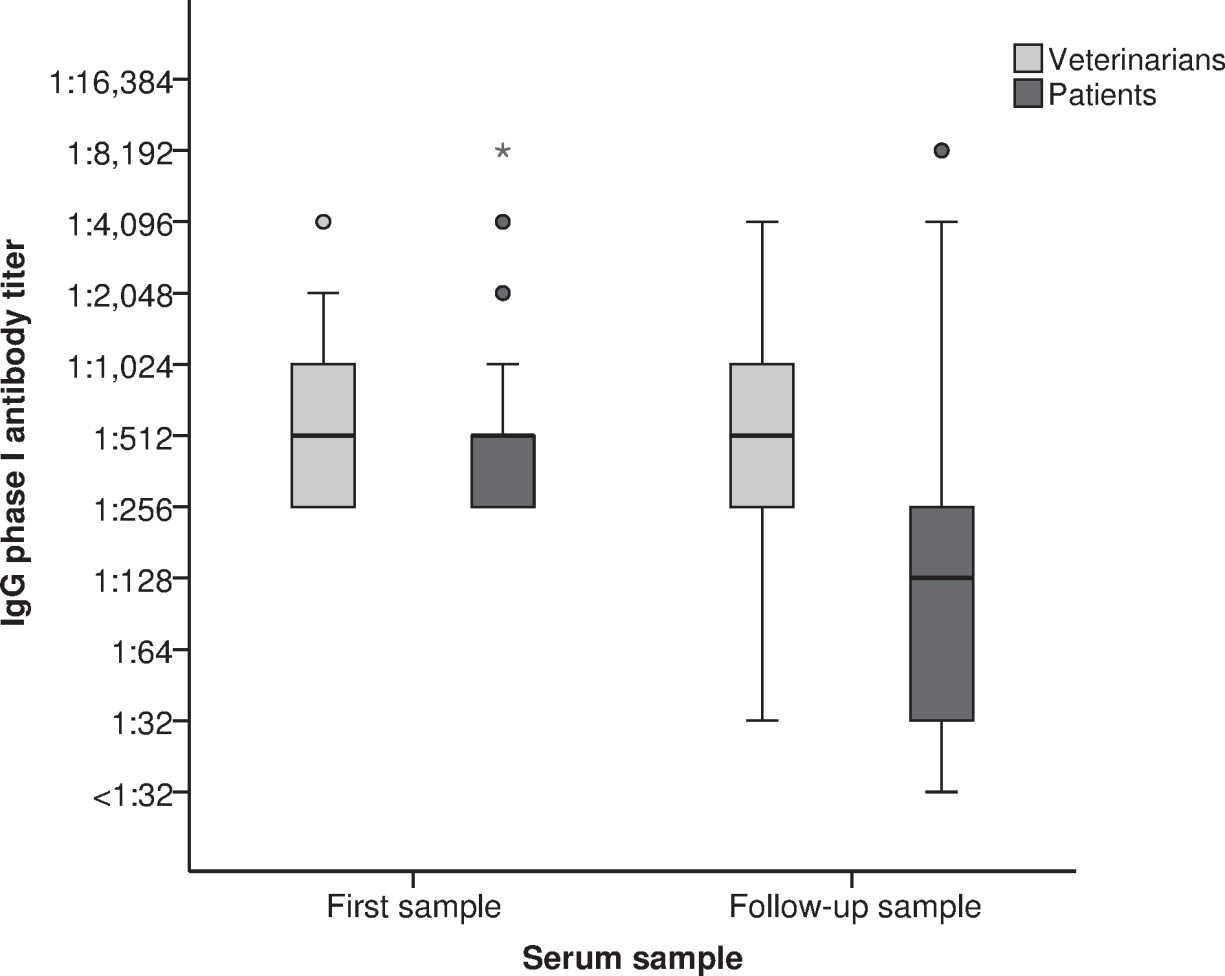
Boxplot of IgG phase I antibodies in two samples obtained from veterinarians (n = 78) and Q fever patients (n = 98) in a three-year time period. The horizontal dark lines within the boxes represent the median antibody titer, the lower and upper boundaries of the boxes represent the 25^th^ and 75^th^ percentiles, and the T-bars represent the 2.5^th^ and 97.5^th^ percentiles. Outliers are indicated with dots, extreme outliers (more than three times the height of the box) with asterisks. First serum sample: veterinarians in 2009 or 2010; patients in 2008 or 2009 (twelve months after acute Q fever diagnosis in 2007 or 2008). Follow-up sample: veterinarians in 2013 (three to four years after first sample); patients in 2011 or 2012 (four years after acute Q fever diagnosis in 2007 or 2008). When no end titration available: >1:2,048 categorized as 1:4,096, and >1:4,096 categorized as 1:8,912.

Sera of all veterinarians were positive for IgG phase I and II antibodies (Table 2). IgG phase I was positive in 83/98 (84.7%) of the Q fever patients, while IgG phase II was positive in all except one patient (99.0%). Veterinarians had significantly higher IgG phase I antibody titers at follow-up and higher IgG phase II titers at the first sample and at follow-up than the Q fever patients (Mann-Whitney U test: *p*<0.001). All *C. burnetii* PCR tests performed had a negative result. The majority of Q fever patients showed decreasing IgG phase I titers, while in veterinarians the majority remained constant or showed a slight increase in IgG phase I titer after a three-year time period (Table 3 and Fig. 1). An IgG phase I titer ≥1:1,024 was found in 27 veterinarians (35.5%) compared to 12 patients (12.2%) in the three-year follow-up sample (OR: 3.95, 95% CI: 1.84–8.49).

**Table 2:** Serological results of IgG phase I and phase II antibody titers in veterinarians (n = 76) and Q fever patients (n = 98) in two samples taken in a three-year time period.

**IgG antibody titer**	**IgG phase I first sample** ^a^		**IgG phase I follow-up** ^b^		**IgG phase II first sample** ^a^		**IgG phase II follow-up** ^b^	
	**Veterinarians (n = 76)**	**Patients (n = 98)**	**Veterinarians (n = 76)**	**Patients (n = 98)**	**Veterinarians (n = 76)**	**Patients (n = 98)**	**Veterinarians (n = 76)**	**Patients (n = 98)**
	**n (%)**	**n (%)**	**n (%)**	**n (%)**	**n (%)**	**n (%)**	**n (%)**	**n (%)**
<1:32	NA	NA	0 (0.0)	15 (15.3)	0 (0.0)	0 (0.0)	0 (0.0)	1 (1.0)
1:32	NA	NA	1 (1.3)	12 (12.2)	1 (1.3)	0 (0.0)	2 (2.6)	1 (1.0)
1:64	NA	NA	1 (1.3)	19 (19.4)	0 (0.0)	0 (0.0)	0 (0.0)	1 (1.0)
1:128	NA	NA	9 (11.8)	21 (21.4)	3 (3.9)	0 (0.0)	4 (5.3)	1 (1.0)
1:256	38 (50.0)	40 (40.8)	18 (23.7)	11 (11.2)	19 (25.0)	0 (0.0)	16 (21.1)	16 (16.3)
1:512	19 (25.0)	36 (36.7)	20 (26.3)	8 (8.2)	37 (48.7)	4 (4.1)	31 (40.8)	13 (13.3)
1:1,024	14 (18.4)	13 (13.3)	21 (27.6)	6 (6.1)	10 (13.2)	18 (18.4)	18 (23.7)	23 (23.5)
1:2,048	4 (5.3)	4 (4.1)	5 (6.6)	3 (3.1)	4 (5.3)	25 (25.5)	4 (5.3)	24 (24.5)
1:4,096	1 (1.3)^d^	2 (2.0)	1 (1.3)	1 (1.0)	2 (2.6)^d^	30 (30.6)	1 (1.3)	14 (14.3)
1:8,192	0 (0.0)	3 (3.1)	0 (0.0)	2 (2.0)^e^	0 (0.0)	21 (21.4)^f^	0 (0.0)	3 (3.1)^g^
1:16,384	0 (0.0)	0 (0.0)	0 (0.0)	0 (0.0)	0 (0.0)	0 (0.0)	0 (0.0)	1 (1.0)
*p*-value^c^	0.419		<0.001		<0.001		<0.001	

NA: not applicable.

^a^ First serum sample: veterinarians in 2009 or 2010; patients in 2008 or 2009 (twelve months after acute Q fever diagnosis in 2007 or 2008).

^b^ Follow-up sample: veterinarians in 2013 (three to four years after first sample); patients in 2011 or 2012 (four years after acute Q fever diagnosis in 2007 or 2008).

^c^ *P*-value calculated with Mann-Whitney U test by comparing antibody titers between veterinarians and Q fever patients.

^d^ One sample with a result of >1:2,048.

^e^ Including one sample with a result of >1:4,096.

^f^ Including 17 samples with a result of >1:4,096.

^g^ Including two samples with a result of >1:4096.

**Table 3:** Course of IgG phase I and phase II antibody titers (increase, constant, decrease) in veterinarians (n = 76) and Q fever patients (n = 98) in two samples taken in a three-year time period.^a^

**Course**		**IgG phase I titer**		**IgG phase II titer**	
		**Veterinarians (n = 76)**	**Patients (n = 98)**	**Veterinarians (n = 76)**	**Patients (n = 98)**
		**n (%)**	**n (%)**	**n (%)**	**n (%)**
Increase	Total increase	28 (36.8)	6 (6.1)	23 (30.3)	6 (6.1)
	2-fold	21 (27.6)	4 (4.1)	17 (22.4)	4 (4.1)
	4-fold	7 (9.2)	0 (0.0)	6 (7.9)	0 (0.0)
	8-fold	0 (0.0)	1 (1.0)	0 (0.0)	0 (0.0)
	≥16-fold	0 (0.0)	0 (0.0)	0 (0.0)	0 (0.0)
	Undetermined^b^	0 (0.0)	1 (1.0)	0 (0.0)	2 (2.0)
Constant		23 (30.3)	10 (10.2)	30 (39.5)	16 (16.3)
Decrease	Total decrease	24 (31.6)	82 (83.7)	23 (30.2)	75 (76.5)
	2-fold	18 (23.7)	17 (17.3)	19 (25.0)	27 (27.6)
	4-fold	5 (6.6)	29 (29.6)	2 (2.6)	17 (17.3)
	8-fold	1 (1.3)	12 (12.2)	1 (1.3)	10 (10.2)
	≥16-fold	0 (0.0)	24 (24.5)	0 (0.0)	7 (7.1)
	Undetermined^b^	0 (0.0)	0 (0.0)	1 (1.3)	14 (14.3)
No end titration, not specified^b^		1 (1.3)	0 (0.0)	0 (0.0)	1 (1.0)

^a^ First serum sample: veterinarians in 2009 or 2010; patients in 2008 or 2009 (twelve months after acute Q fever diagnosis in 2007 or 2008). Follow-up sample: veterinarians in 2013 (three to four years after first sample); patients in 2011 or 2012 (four years after acute Q fever diagnosis in 2007 or 2008).

^b^ Because some samples did not have and end titration (e.g., >1:4,096), the number of decreased dilutions could not be established.

The number of veterinarians mainly working with livestock was 52 (68.4%) while the other 24 (31.6%) mainly worked with companion animals.The majority of the veterinarians with IgG phase I ≥1:1,024 was a livestock veterinarian (22/27; 81.5%). Livestock veterinarians had higher IgG phase I titers than the companion animal veterinarians (Mann-Whitney U test: *p* = 0.06 in first sample and *p* = 0.02 at three-year follow-up). None of the veterinarians reported a risk factor for chronic Q fever (aneurysm, vascular or valvular prosthesis or disease) in the administered questionnaire.

None of the potential risk factors for which information was available (living region, hours of animal contact, contact with different types of livestock or companion animals or birth products and symptoms of acute Q fever in medical history) showed an association with increasing or constant IgG phase I antibody titers.

## Discussion

After a serological survey with an initial IgG phase I titer ≥1:256, 27 (35.5%) veterinarians had an IgG phase I titer ≥1:1,024 three years later, and could therefore be classified as ‘possible chronic Q fever’ according to the criteria of the Dutch Q fever Consensus Group [11]. These veterinarians were advised to visit a clinician to detect possible symptoms and risk factors for chronic Q fever according to the recommendations of this consensus group.

In general, we observed that the IgG phase I titers among veterinarians remained constant or showed a slight increase, while the large majority of Q fever patients showed a decreasing trend in IgG phase I levels during three-year follow-up. Also, veterinarians had significantly higher IgG phase I titers at three-year follow-up than Q fever patients. Usually, when people are not repeatedly exposed to a pathogen, antibody titers will decay over time [23]. Our hypothesis on this observation of constant or slightly increased antibody titers among veterinarians is that they, and possibly also other related occupational groups, are frequently exposed to *C. burnetii* and that therefore the antibody levels do not show the natural decay. This continued exposure, however, causes a boostering effect. This has also been suggested by Wattiau *et al*., who described antibody profiles of wool workers in Belgium with an increased IgG phase II, IgM phase II or both, which suggests re-infection or repeated stimulation of the immune response due to continuous exposure to *C. burnetii* [24]. This boostering effect in occupationally exposed persons possibly leads to protective immunity and protects against symptomatic illness, which has also been described for *Campylobacter*: an infection with *Campylobacter* can remain asymptomatic and it is suggested that exposure to *Cambylobacter* may lead to transient protective immunity and protects against symptomatic illness [25–27].

Despite these high seroprevalences, the number of reported chronic Q fever infections among occupationally exposed persons in the literature is low. This is also due to the fact that veterinarians form a relatively small occupational group (3,249 practicing veterinarians in 2013 in the Netherlands, numbers provided by De Koninklijke Nederlandse Maatschappij voor Diergeneeskunde (KNMvD), The Royal Dutch Society of Veterinary Medicine). In contrary, the proportion of chronic Q fever among this relatively small occupational group might be higher. However, there are no longitudinal observational studies among occupational exposed people on symptoms of chronic Q fever.

Whether a high IgG phase I titer (≥1:1,024) of *C. burnetii* antibodies without further signs of a chronic infection has clinical significance is unclear, but serious health consequences are unlikely in persons without risk factors for a chronic infection based on epidemiological experiences. In the study performed by Bosjnak *et al.* in Denmark, 11 of the 50 seropositive occupationally exposed persons had an IgG phase I titer ≥1:1,024 [28]. Signs and symptoms of a chronic infection were only found in one case, and according to the criteria of the Dutch Q fever Consensus Group, this case would be classified as probable chronic Q fever [28]. Wattiau *et al.* reported that two wool workers had serological evidence for a chronic Q fever infection, but clinical examinations and the *C. burnetii* PCR test were all negative and no symptoms were present. The same observation, but with a shorter follow-up period of four to eight months, was described in German veterinarians by Bernard *et al*.: in 17/424 (4%) participating veterinarians chronic Q fever could not be excluded initially (with IgG phase I ≥1:512 used as cut-off) [18].

Because of the persistence of IgG phase I titers, serological and medical follow-up should be considered for veterinarians, and other occupationally exposed groups, with IgG phase titers ≥1:1,024. During a medical evaluation, risk factors for chronic Q fever need to be excluded. Occupational physicians need to be aware of different categories of chronic Q fever and of the fact that elevated IgG phase I titers among occupationally exposed employees may reflect a boostering effect rather than chronic Q fever. Occupational physicians can play a role in requesting diagnostic and medical follow-up.

The present study has a few limitations. First, the determination of the antibody titers by using IFA is subjective and therefore sensitive to interobserver bias, and serum samples from the previous and the current study were not tested simultaneously. However, all samples of the veterinarians as well as the patients were tested in the same accredited laboratory, which also participates in quality checks. Furthermore, this regional laboratory was located in the epidemic Q fever area and enormous numbers of samples have been analyzed with the IFA test.

Secondly, the current study is based on two measurements with an interval of approximately three years in veterinarians and Q fever patients. This period, however, differed significantly between veterinarians and Q fever patients, although it has been shown that especially IgG phase II titers show a slow decay [23]. The exact moment of the primary *C. burnetii* infection in veterinarians is unknown, because no date of onset of symptoms is known or the infection remained asymptomatic. It is therefore uncertain whether the follow-up period between the two collected serum samples in veterinarians is comparable to the follow-up period in the patients that were symptomatic due to a recent acute Q fever episode. As the Q fever epidemic occurred in the Netherlands between 2007 and 2010, it is plausible that livestock veterinarians were exposed to *C. burnetii* due to direct contact with infected small ruminant herds and/or through living or working in the Q fever affected area. However, it has been shown that there were *C. burnetii* infected goat farms in the Netherlands before the start of the epidemic in 2007 [29], and it is likely that *C. burnetii* has been present in cattle already for a long time, without leading to a significant number of symptomatic infections in humans [30]. Therefore, the infection among veterinarians might also have occurred before the start of the Dutch epidemic.

Thirdly, the data of the veterinarians working in clinical practice were collected in 2009 and 2010. It is therefore unknown whether the veterinarians participating in this follow-up study were still practicing or not. The final limitation is that the risk factors for a chronic Q fever infection were self-reported.

In conclusion, this study demonstrates that IgG phase I antibodies against *C. burnetii* remain high in the majority of the veterinarians in a three-year time period compared with patients who were diagnosed with acute Q fever four years earlier. To our knowledge, this is the first follow-up study among veterinarians which describes antibody titers measured over period of three years and compares these results with non-occupationally exposed people. According to the Dutch Q fever Consensus Group criteria, 27 veterinarians that participated in this study were classified as having possible chronic Q fever, of whom 22 were livestock veterinarian. Continuous exposure to *C. burnetii* is a likely cause of the elevated IgG phase I antibody responses in absence of reported clinical symptoms. Given the current uncertainties about the development of chronic Q fever among occupationally exposed people, such as veterinarians, serological and medical follow-up of this group with risk factors for chronic Q fever should be considered.

## References

[1] Maurin M, Raoult D (1999) Q fever. Clin Microbiol Rev 12: 518–553. 10515901 10.1128/cmr.12.4.518 PMC88923

[2] Parker NR, Barralet JH, Bell AM (2006) Q fever. Lancet 367: 679–688. 16503466 10.1016/S0140-6736(06)68266-4

[3] Raoult D, Marrie TJ, Mege JL (2005) Natural history and pathophysiology of Q fever. Lancet Infect Dis 5: 219–226. 15792739 10.1016/S1473-3099(05)70052-9

[4] van der Hoek W, Dijkstra F, Schimmer B, Schneeberger PM, Vellema P, et al. (2010) Q fever in the Netherlands: an update on the epidemiology and control measures. Euro Surveill 15: pii = 19520. 20350500

[5] van der Hoek W, Schneeberger PM, Oomen T, Wegdam-Blans MC, Dijkstra F, et al. (2012) Shifting priorities in the aftermath of a Q fever epidemic in 2007 to 2009 in the Netherlands: from acute to chronic infection. Euro Surveill 17: pii:20059. 22297101

[6] Angelakis E, Raoult D (2010) Q fever. Vet Microbiol 140: 297–309. 19875249 10.1016/j.vetmic.2009.07.016

[7] Kampschreur LM, Delsing CE, Groenwold RH, Wegdam-Blans MC, Bleeker-Rovers CP, et al. (2014) Chronic Q fever in the Netherlands five years after the start of the Q fever epidemic: results from the Dutch Chronic Q Fever Database. J Clin Microbiol 52: 1637–1643. 10.1128/JCM.03221-13 24599987 PMC3993626

[8] European Centre for Disease Prevention and Control (ECDC) (2010) Risk assessment on Q fever. Stockholm: ECDC 10.2900/28860.

[9] Fenollar F, Fournier PE, Carrieri MP, Habib G, Messana T, et al. (2001) Risks factors and prevention of Q fever endocarditis. Clin Infect Dis 33: 312–316. 11438895 10.1086/321889

[10] Kampschreur LM, Dekker S, Hagenaars JCJP, Lestrade PJ, Renders NHM, et al. (2012) Identification of risk factors for chronic Q fever, the Netherlands. Emerg Infect Dis 18: 563–570. 22469535 10.3201/eid1804.111478 PMC3309671

[11] Wegdam-Blans MCA, Kampschreur LM, Delsing CE, Bleeker-Rovers CP, Sprong T, et al. (2012) Chronic Q fever: review of the literature and a proposal of new diagnostic criteria. J Infect 64: 247–259. 10.1016/j.jinf.2011.12.014 22226692

[12] Raoult D (2012) Chronic Q fever: Expert opinion versus literature analysis and consensus. J Infect 65: 102–108. 10.1016/j.jinf.2012.04.006 22537659

[13] Kampschreur LM, Wever PC, Wegdam-Blans MCA, Delsing CE, Bleeker-Rovers CP, et al. (2012) Defining chronic Q fever: a matter of debate. J Infect 65: 362–363. 10.1016/j.jinf.2012.08.002 22898388

[14] van den Brom R, Schimmer B, Schneeberger PM, Swart WA, van der Hoek W, et al. (2013) Seroepidemiological survey for *Coxiella burnetii* antibodies and associated risk factors in Dutch livestock veterinarians. PLoS One 8: e54021. 23342063 10.1371/journal.pone.0054021 PMC3546960

[15] de Lange MM, Schimmer B, Vellema P, Hautvast JLA, Schneeberger PM, et al. (2014) *Coxiella burnetii* seroprevalence and risk factors in sheep farmers and farm residents in The Netherlands. Epidemiol Infect: 142: 1231–1244. 23920311 10.1017/S0950268813001726 PMC4045170

[16] Schimmer B, Lenferink A, Schneeberger P, Aangenend H, Vellema P, et al. (2012) Seroprevalence and risk factors for *Coxiella burnetii* (Q fever) seropositivity in dairy goat farmers’ households in The Netherlands, 2009–2010. PLoS One 7: e42364 10.1371/journal.pone.0042364 22848762 PMC3407076

[17] Whitney EAS, Massung RF, Candee AJ, Ailes EC, Myers LM, et al. (2009) Seroepidemiologic and occupational risk survey for *Coxiella burnetii* antibodies among US veterinarians. Clin Infect Dis 48: 550–557. 10.1086/596705 19191638

[18] Bernard H, Brockmann SO, Kleinkauf N, Klinc C, Wagner-Wiening C, et al. (2012) High seroprevalence of *Coxiella burnetii* antibodies in veterinarians associated with cattle obstetrics, Bavaria, 2009. Vector Borne Zoonotic Dis 12: 552–557. 10.1089/vbz.2011.0879 22607080

[19] EuroQol Group (1990) EuroQol--a new facility for the measurement of health-related quality of life. Health Policy 16: 199–208. 10109801 10.1016/0168-8510(90)90421-9

[20] Peters JB, Daudey L, Heijdra YF, Molema J, Dekhuijzen PN, et al. (2009) Development of a battery of instruments for detailed measurement of health status in patients with COPD in routine care: the Nijmegen Clinical Screening Instrument. Qual Life Res 18: 901–912. 10.1007/s11136-009-9502-2 19543807 PMC2724638

[21] Schneeberger PM, Hermans MHA, van Hannen EJ, Schellekens JJA, Leenders ACAP, et al. (2010) Real-time PCR with serum samples is indispensable for early diagnosis of acute Q fever. Clin Vaccine Immunol 17: 286–290. 10.1128/CVI.00454-09 20032219 PMC2815520

[22] Wielders CCH, Wijnbergen PCA, Renders NHM, Schellekens JJA, Schneeberger PM, et al. (2013) High *Coxiella burnetii* DNA load in serum during acute Q fever is associated with progression to a serologic profile indicative of chronic Q fever. J Clin Microbiol 51: 3192–3198. 10.1128/JCM.00993-13 23863573 PMC3811622

[23] Teunis PFM, Schimmer B, Notermans DW, Leenders ACAP, Wever PC, et al. (2013) Time-course of antibody responses against *Coxiella burnetii* following acute Q fever. Epidemiol Infect 141:62–73. 10.1017/S0950268812000404 22475210 PMC9152070

[24] Wattiau P, Boldisova E, Toman R, Van Esbroeck M, Quoilin S, et al. (2011) Q fever in Woolsorters, Belgium. Emerg Infect Dis 17: 2368–2369. 10.3201/eid1712.101786 22172399 PMC3311209

[25] Swart AN, Tomasi M, Kretzschmar M, Havelaar AH, Diekmann O (2012) The protective effects of temporary immunity under imposed infection pressure. Epidemics 4: 43–47. 10.1016/j.epidem.2011.12.002 22325013

[26] Teunis P, Van den Brandhof W, Nauta M, Wagenaar J, Van den Kerkhof H, et al. (2005) A reconsideration of the *Campylobacter* dose-response relation. Epidemiol Infect 133: 583–592. 16050502 10.1017/s0950268805003912 PMC2870284

[27] Teunis PFM, Falkenhorst G, Ang CW, Strid MA, De Valk H, et al. (2013) *Campylobacter* seroconversion rates in selected countries in the European Union. Epidemiol Infect 141: 2051–2057. 10.1017/S0950268812002774 23228443 PMC9151417

[28] Bosnjak E, Hvass AMSW, Villumsen S, Nielsen H (2010) Emerging evidence for Q fever in humans in Denmark: role of contact with dairy cattle. Clin Microbiol Infect 16: 1285–1288. 10.1111/j.1469-0691.2009.03062.x 19832723

[29] van den Wijngaard CC, Dijkstra F, van Pelt W, van Asten L, Kretzschmar M, et al. (2011) In search of hidden Q-fever outbreaks: linking syndromic hospital clusters to infected goat farms. Epidemiol Infect 139: 19–26. 10.1017/S0950268810001032 20478085

[30] Roest HIJ, Tilburg JJHC, van der Hoek W, Vellema P, van Zijderveld FG, et al. (2011) The Q fever epidemic in The Netherlands: history, onset, response and reflection. Epidemiol Infect 139: 1–12. 10.1017/S0950268810002268 20920383

